# Depletion of the TRF1 telomere-binding protein leads to leaner mice with altered metabolic profiles

**DOI:** 10.18632/aging.206320

**Published:** 2025-09-17

**Authors:** Jessica Louzame Ruano, Leire Bejarano, Rosa Serrano, Ruth Alvarez Diaz, Juana Maria Flores, Ana Cayuela López, Maria A. Blasco

**Affiliations:** 1 Telomeres and Telomerase Group, Molecular Oncology Program, Spanish National Cancer Centre (CNIO), Madrid E-28029, Spain; 2 Bioinformatics Unit, Spanish National Cancer Centre (CNIO), Madrid E-28029, Spain; 3 Animal Surgery and Medicine Department, Faculty of Veterinary Science, Complutense University of Madrid, Madrid, Spain; 4 Confocal Microscopy Unit, Spanish National Cancer Centre (CNIO), Madrid E-28029, Spain

**Keywords:** Trf1, metabolism, leaner, fat, telomeres

## Abstract

TRF1, a component of the telomere shelterin complex, plays crucial roles in telomere protection, telomere length regulation, and stemness. Here, we describe a previously unknown connection between TRF1 and metabolism. Telomere attrition has been linked to obesity. Our study reveals that *Trf1*-deficient mice exhibit a leaner phenotype, reduced adiposity, and improved glucose tolerance, even when subjected to a high-fat diet, independently of telomere shortening. These findings uncover a previously unknown role of TRF1 in regulating metabolism.

## INTRODUCTION

Telomeres are specialized protective structures located at the ends of chromosomes that are composed of tandem repeats of the DNA TTAGGG sequence in all vertebrate species. Telomeres act as protective caps, preventing degradation of chromosome ends and chromosome aberrations, such as end-to-end chromosome fusions. Telomeres shorten throughout the lifespan of individuals associated with cell renewal and proliferation owing to the so-called “end replication problem”. Thus, telomeres become progressively shorter until they reach a critically short length, which triggers a persistent DNA damage response at telomeres leading to cell arrest or apoptosis [1], thus impairing tissue homeostasis. Short telomeres have been considered one of the primary hallmarks of aging as they can trigger other molecular and cellular events associated with aging [2, 3].

Telomerase is a reverse transcriptase enzyme that can extend telomeres by adding *de novo* TTAGGG sequences. Telomerase is composed of a catalytic subunit (TERT, telomerase reverse transcriptase) and an RNA component (Terc, telomerase RNA component), which serves as template for the addition of new telomeric repeats [4]. Telomeres are protected by the so-called Shelterin complex, which encompasses TRF1, TRF2, TIN2, TPP1, RAP1, and POT1 proteins [5–7]. TRF1 directly binds to the double-stranded telomere DNA repeats and plays a crucial role in protecting and stabilizing telomeres. Additionally, TRF1 functions as a negative regulator for telomere length by inhibiting telomerase activity [8–10]. Furthermore, TRF1 is implicated in the maintenance of pluripotency [6, 11–13] and, it is overexpressed in induced pluripotent stem cells as well as adult stem cells, including cancer stem cells [14].

Obesity is one of the leading causes of death worldwide and its frequency increased with demographic aging, suggesting a link between the age-associated development of obesity and the molecular mechanisms of biological aging [2, 3]. In fact, short and dysfunctional telomeres have been associated with metabolic disorders such as obesity or diabetes mellitus [15, 16]. Since short telomeres are a known hallmark of aging and they have been demonstrated to be one of the primary causes of organismal aging [2, 3]. A previous study found that young late-generation (G4) *Terc*-deficient mice with short telomeres exhibited defective glucose tolerance and impaired insulin secretion due to the progressive failure of pancreatic beta-cell regeneration leading to the loss of these cells which constitutes a hallmark of type 2 diabetes [17]. Recently, it was described the effect of short telomeres in adipose progenitor cells in *Tert*-deficient mice, which led to adipocyte hypertrophy, fibrosis and inflammation in visceral and subcutaneous adipose tissue resulting in acceleration of metabolic disease [18]. Furthermore, telomere dysfunction has been shown to activate p53 which represses the expression of *PGC1a* and *PGC1b*, impairing mitochondrial respiration, biogenesis and function resulting in decreased gluconeogenesis, cardiomyopathy and increased reactive oxygen species [19]. In turn, mice born with hyper-long telomeres are significantly leaner than control mice and show clear metabolic improvements, such as improved glucose and insulin tolerance [20]. Moreover, we and others also showed that mice lacking the telomere-binding protein RAP1 develop obesity and metabolic syndrome as the consequence of an altered transcription of the Pparα/Pgc1 axis [21, 22]. Finally, in a human cohort it was described the role of TRF1 as a major regulator of telomere attrition in patient with obesity [23].

Here, we show the mice lacking TRF1 are leaner than wild-type controls owing to decreased adiposity. TRF1 deficiency leads to improved glucose and insulin tolerance and protects from metabolic syndrome. These results show an unprecedented role of TRF1 in metabolism.

## RESULTS

### Mice lacking TRF1 in the whole organism show decreased body weight and adiposity

**Figure 1:**
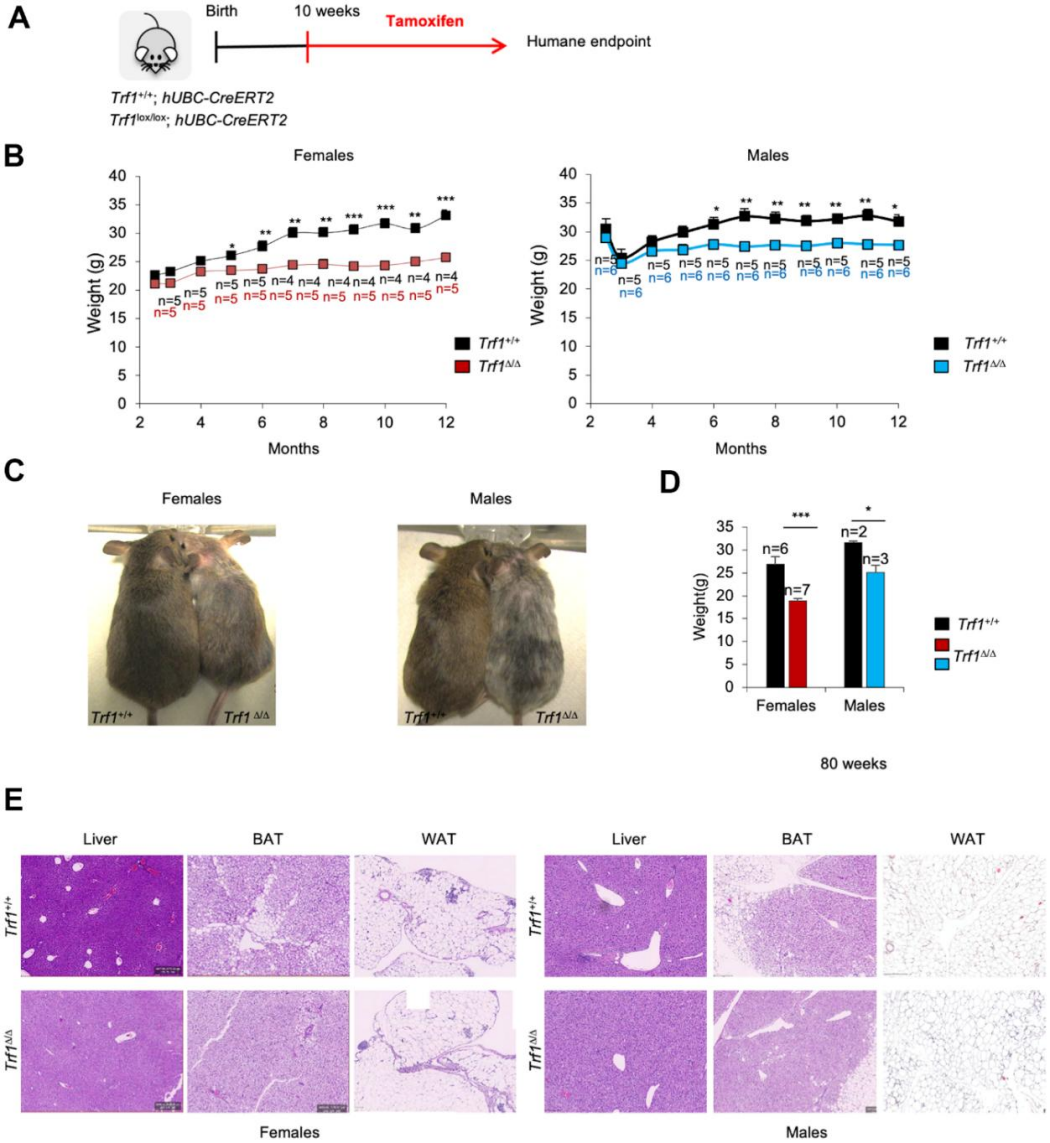
**Depletion of *Trf1* in mice induces weight loss**. (**A**) Experimental plan: *Trf1^+/+^* or *Trf1^lox/lox^*; hUBC-CreERT2 mice start to receive tamoxifen treatment intraperitoneally at 10 weeks of age until humane endpoint. (**B**) Weight follow-up in females (left) and males (right) in both genotypes. Note that *Trf* *^Δ/Δ^* females start to weigh less than wild-type at five months and males at six months. (**C**) Representative images of wild-type and *Trf1* deleted mice of 10 months of age. Note the observable difference in weight and graying hair in *Trf1^Δ/Δ^* mice. (**D**) Measurement of body weight in 80-week-old mice. Note that the difference in weight is maintained throughout their lifespan. (**E**) Hematoxylin and eosin staining of liver, white and brown adipose tissue. Note that there are no differences between genotypes regarding liver and white adipose tissue. In brown adipose tissue, *Trf1^Δ/Δ^* mice present fewer and smaller lipid droplets than wild-type mice. Error bars, s.e.m.; only significant values are shown; *P < 0.05; **P < 0.01; ***P < 0.001 determined by two-tailed Student’s *t*-test (**B**, **D**)

We previously showed that deleting the *Trf1* gene in both *wild-type* and cancer-prone (due to *p53* and *Ink4Arf* deficiencies) mouse models had no significant impact on mouse survival, although in some genetic backgrounds led to reduced body weight and mild hair and skin phenotypes [24]. To gain a deeper understanding on the weight reduction associated to TRF1 abrogation in mice, here we induced persistent *Trf1* deletion from ten weeks of age until humane endpoint in *Trf1*^lox/lox^, *hUBC-CreERT2* mice by intraperitoneal injection of tamoxifen (Figure 1A). Consistent with previous observations, no significant differences in survival were found between genotypes (Supplementary Figure 1A). Interestingly, we observed a significantly decreased body weight starting at five months of age in *Trf1^Δ/Δ^* females and at six months of age in *Trf1^Δ/Δ^* males compared to age-matched wild-type controls (Figure 1B, 1C). The decreased body weight was maintained throughout their remaining lifespan (Figure 1D).

*Trf1* is a target of the pluripotency factor OCT4 and has been previously shown by us to play a role in maintenance of pluripotency, as well as stemness [14]. Given the high proliferative capacity of the intestine, we investigated whether the reduced body weight observed in *Trf1*-deleted mice could be the result of intestinal atrophy owing to TRF1 abrogation. To this end, we measured the length of their intestinal villi. We did not observe any significant differences in villi length between wild-type and *Trf1*-deleted mice in both genders (Supplementary Figure 1B). Next, a comprehensive histological analysis of mice of both genotypes and sexes at the time of death was performed by a pathologist to assess the potential effects of *Trf1* deletion in the organism which could explain the observed differences in body weight. However, we did not detect any differences between genotypes in pancreas, spleen, intestin, colon, kidney, heart, lung, brain and muscle. We next further explored metabolic organs, such as liver, as well as brown and white adipose tissues. We did not observe any differences between genotypes in liver or white adipose tissue. Of interest, the brown adipose tissue of *Trf1* depleted mice exhibited fewer lipid droplets compared to wild-type controls. (Figure 1E).

Previous studies have linked telomere shortening with metabolic syndrome [17–19, 23]. Thus, we set to address whether TRF1 deficiency in mice leads to telomere shortening. To this end, we performed quantitative telomere fluorescence hybridization (Telomere Q-FISH). However, we did not detect any differences in telomere length between genotypes in the liver as well as the white adipose and brown adipose tissue (Supplementary Figure 1C). Consistent with prior studies reported, no change in telomere length upon TRF1 depletion (Piñeiro-Hermida et al., 2022; Bejarano et al., 2017).

To further investigate the origin of the decreased body weight of *Trf1^Δ/Δ^* mice, we next determined total fat content in 40 weeks old males and females by using Dual-energy X-ray absorptiometry (DEXA). We found a striking reduction in fat mass and adiposity in both *Trf1^Δ/Δ^* males and females compared to wild-type controls, which was maintained at 80 weeks of age.

**Figure 2:**
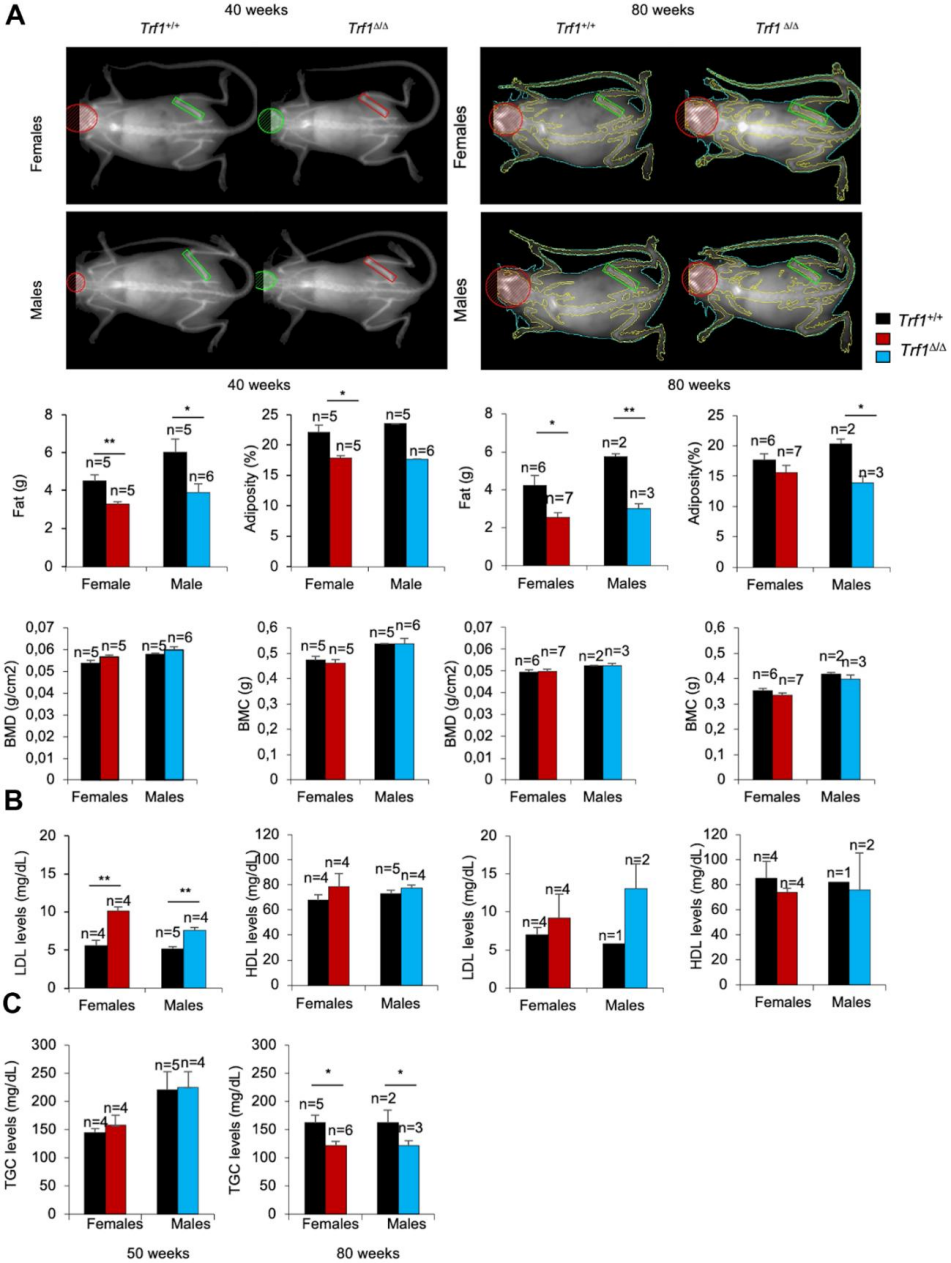
**The fat content is reduced in *Trf1****^Δ/Δ^* **mice**. (**A**) Top: representative images of *Trf1^+/+^* and *Trf1^Δ/Δ^* females and males obtained with Dual-energy X-ray absorptiometry (DEXA). Bottom: Quantification of fat (g), adiposity (%), bone mineral density (g/cm^2^) and bone mineral composition (g) measured obtained by Dual-energy X-ray absorptiometry (DEXA) at 40 and 80 weeks respectively. Note that *Trf1^Δ/Δ^* have less adiposity and fat content compared to wild-types. (**B**) LDL and HDL levels in serum 40- and 80- week-old-mice of the indicated gender and genotype. *Trf1^Δ/Δ^* of 40 weeks mice exhibited more LDL levels (mg/dL) in both sexes. (**C**) Triglycerides levels in serum in 50 and, 80 weeks old mice of both genotype and gender. Note that older (80 weeks) *Trf1^Δ/Δ^* mice had lower TGC (triglycerides) levels in serum compared to wild-types. Error bars, s.e.m.; only significant values are shown; *P < 0.05; **P < 0.01; ***P < 0.001 determined by two-tailed Student’s *t*-test (**A**–**C**)

Bone mineral density and bone mineral content, however, remained similar between genotypes (Figure 2A). Thus, the differences in body weight can be attributed to reduced fat content as the consequence of TRF1 abrogation.

Next, we set to measure several metabolic parameters in blood. Of note, LDL cholesterol levels were significantly higher in 40 weeks old *Trf ^Δ/Δ^* male and female mice compared to wild-type controls, while plasma HDL cholesterol levels remained similar (Figure 2B).

This trend persisted at 80 weeks of age; however, differences in both LDL and HDL levels did not reach statistical significance (Figure 2B). Plasma triglycerides levels were similar in 50 weeks old *Trf1^Δ/Δ^* and wild-type mice but were significantly decreased at 80 weeks of age in *Trf1^Δ/Δ^* mice of both genders (Figure 2C). None of the other metabolic and blood chemistry parameters tested showed significant differences between genotypes (Supplementary Figure 1D). Altogether, these findings indicate that *Trf1* deletion induced a persistent weight reduction due to decrease adiposity.

### Mice lacking TRF1 in the whole organism show improved glucose and insulin metabolism with aging

**Figure 3:**
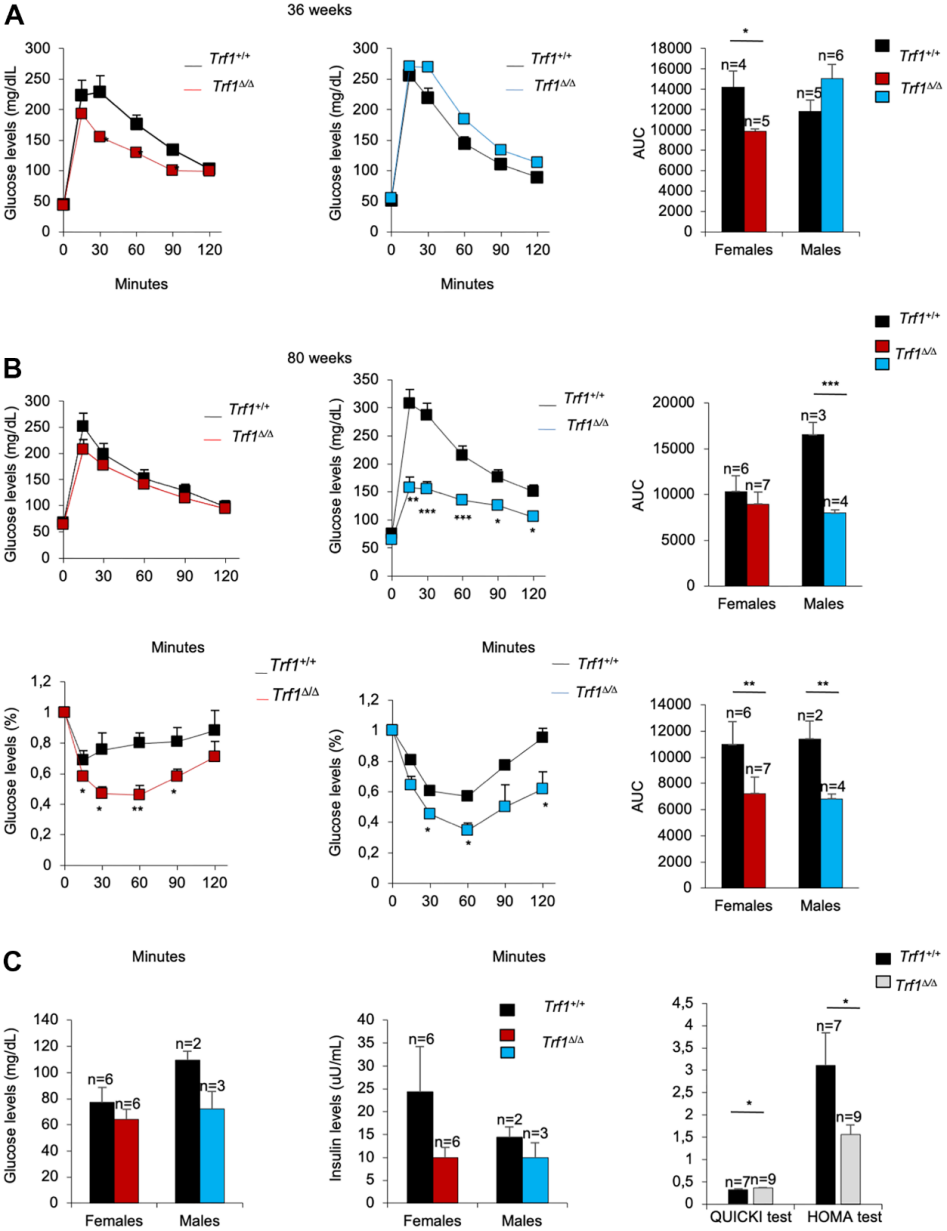
***Trf1^Δ/Δ^* mice show better tolerance to glucose.** (**A**) Glucose tolerance test in 36-week-old-mice. Left: fasting glucose levels (mg/dl) measured at different time points (minutes) in females and males. Right: quantification of the area under the glucose tolerance test curve (AUC) in females and males. Note that *Trf1 ^Δ/Δ^* females have lower glucose levels compared to wild-types. (**B**) Glucose and insulin tolerance test in 80-week-old-mice. Up: Glucose tolerance test. Left: fasting glucose levels (mg/dl) measured at different time points (minutes). Right: quantification of the area under the glucose tolerance test curve (AUC) in females and males. Bottom: Insulin tolerance test. Left: glucose levels (%) relative to glucose levels in fasting measured at different time points (minutes). Right: quantification of the area under the insulin tolerance test curve (AUC) in females and males. Note that *Trf1^Δ/Δ^* males have a better tolerance to glucose than wild-types. In addition, *Trf1^Δ/Δ^* mice showed better tolerance to insulin. (**C**) Fasting glucose and fasting insulin levels, derived HOMA-IR insulin-resistance quantification, and QUICKI insulin sensitivity quantification of 80-week-old males and females. Note that *Trf1^Δ/Δ^* have increased insulin sensitivity and improved insulin resistance shown by QUICKI and HOMA-IR index respectively. Error bars, s.e.m.; only significant values are shown; *P < 0.05; **P < 0.01; ***P < 0.001 determined by two-tailed Student’s *t*-test (**A**–**C**)

Given the unprecedented effect of *Trf1* deletion on reducing fat content and body weight, we next set to address whether *Trf1* abrogation affected other major metabolic parameters, such as glucose and insulin metabolism. At thirty-six weeks of age, *Trf1^Δ/Δ^* mice showed improved glucose tolerance compared to wild-type controls, reaching statistical significance in the case of females (Figure 3A). In turn, at eighty weeks of age, *Trf1^Δ/Δ^* males showed better glucose tolerance compared to wild-type controls. Accordingly, both *Trf1^Δ/Δ^* male and female mice showed an improved tolerance to insulin at eighty weeks of age compared to wild-type mice (Figure 3B). Of note, upon fasting, however, we found similar glucose and insulin levels in both genders at eighty weeks of age. The derived insulin resistance and insulin sensitivity indices, *ho*meostatic *m*odel *a*ssessment (HOMA-IR), and *qu*antitative *i*nsulin sensitivity *c*hec*k i*ndex (QUICKI), respectively, revealed improved insulin resistance and increased insulin sensitivity in *Trf1* deleted mice of both genders at eighty weeks of age (Figure 3C). In summary, *Trf1^Δ/Δ^* mice exhibit an enhanced glucose metabolism with aging.

### Mice lacking TRF1 in the whole organism show normal fitness

**Figure 4:**
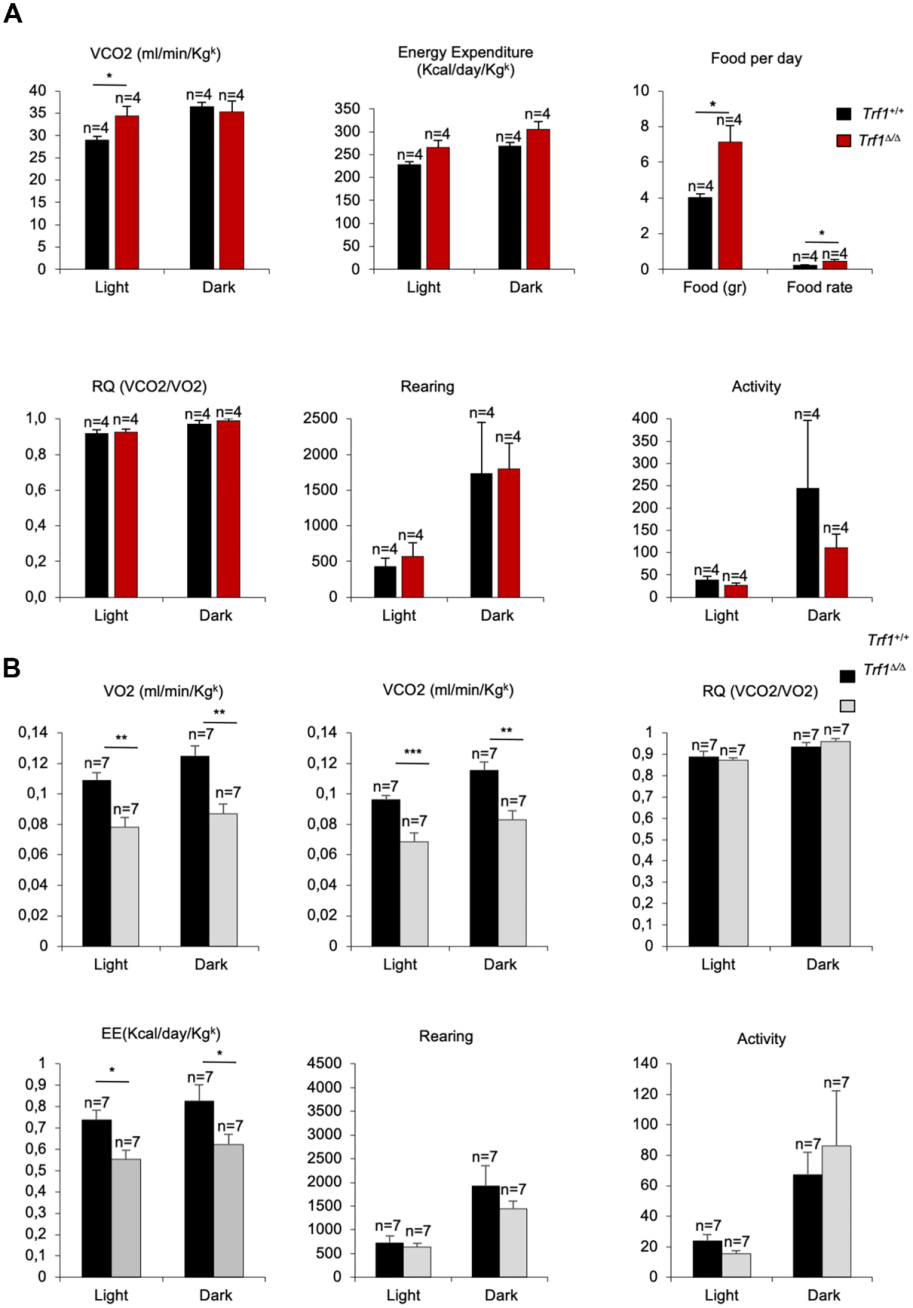
***Trf1^Δ/Δ^* deleted mice show enhanced fitness compared to wild type mice**. (**A**) Volume of dioxide produced (VCO2) (ml/min/Kg^k^), energy expenditure rate (EE) (Kcal/day/Kg^k^), food intake per day, respiratory quotient (RQ), rearing and activity monitorization during light and dark cycles in metabolic cages in 43 weeks females’ mice. Note that females without *Trf1* produce more volume of CO2, tend to consume more energy, and have higher food intake than wild-types. (**B**) Volume of oxygen consumed (VO2), volume of carbon dioxide produced (VCO2) (ml/min/Kg^k^), respiratory quotient (RQ), energy expenditure rate (EE) (Kcal/day/Kg^k^), activity and rearing monitorization during light and dark cycles in 80 weeks females and male’s mice. Note that *Trf1^Δ/Δ^* mice had significantly lower VO2, VCO2, and energy expenditure compared to wild-types. Error bars, s.e.m.; only significant values are shown; *P < 0.05; **P < 0.01; ***P < 0.001 determined by two-tailed Student’s *t*-test (**A**, **B**)

We next determined whether decreased body weight and improved lipid, glucose and insulin metabolism in *Trf1^Δ/Δ^* mice could be attributed to a lower food intake or to an increase physical activity as the consequence of TRF1 protein abrogation. To this end, we subjected forty-three weeks old females to metabolic cages. Due to the limited space, we could not study male mice. We found that *Trf1*-deficient females produce more volume of dioxide (VCO2) and tend to use more energy compared to wild-type females during light cycles. The production of more volume of CO2 and the use of more energy indicates an increase metabolic rate of females without *Trf1* compared to wild-type controls (Figure 4A). Of interest, in spite of their lower weight, *Trf1^Δ/Δ^* mice showed a higher food intake, indicating that the lower body weight is not the consequence of a lower food intake (Figure 4A). We did not find any differences between genotypes in the respiratory quotient (RQ), activity, and rearing (Figure 4A). At eighty-two weeks of age, we found significantly lower levels of VCO2, volume of oxygen (VO2), and energy consumed in *Trf1^Δ/Δ^* mice of both genders compared to wild-types controls (Figure 4B). At this age, no differences between genotypes were observed in activity, rearing and respiratory quotient (Figure 4B). In summary, the leanness observed in *Trf1^Δ/Δ^* mice cannot be attributed to reduced food intake or increased physical activity levels. Interestingly, in forty-three weeks old females, the absence of TRF1 leads to heightened metabolic activity as evidenced by increased production of CO2 volume, higher energy consumption, and elevated food intake compared to wild-type controls.

### Gene expression changes in mice lacking TRF1

**Figure 5:**
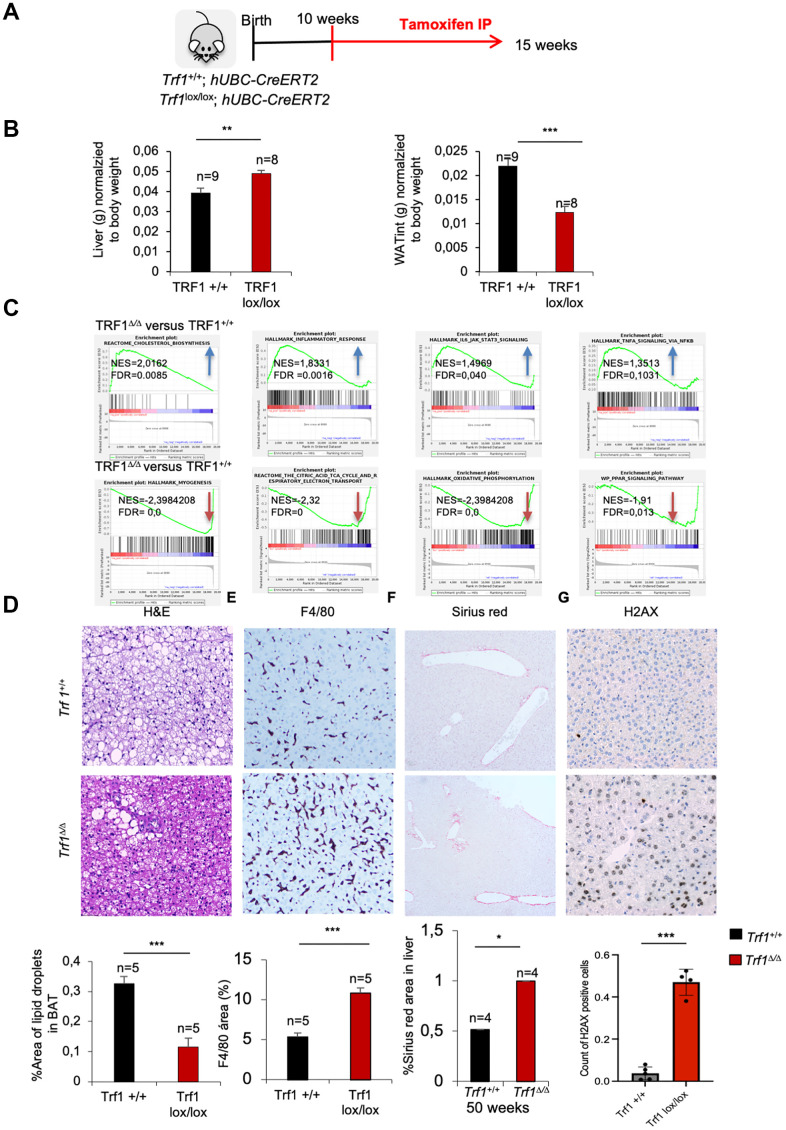
**Deletion of Trf1 induces differences in tissue weight in early stages, before body weight differences**. (**A**) Experimental plan: *Trf1*^+/+^ and *Trf1*^lox/lox^; *hUBC-CreERT2* female mice were treated with tamoxifen at 10 weeks of age. Mice were euthanized 5 weeks after tamoxifen treatment. (**B**) Weight of liver and white adipose tissue (grams) normalized to body weight. Note that *Trf1 ^Δ/Δ^* liver weighted more compared to the wild-types, while the opposite was observed for white adipose tissue. (**C**) Gene expression data obtained by RNA-seq of liver samples of 15 weeks *Trf1*^+/+^ and *Trf1^Δ/Δ^* was analyzed by GSEA to determine significantly enriched gene sets. GSEA plots for the indicated pathways in liver samples for *Trf1^Δ/Δ^* versus *Trf1*^+/+^ mice. The red to blue horizontal bar represents the ranked list. Genes located at the central area of the bar show small differences in gene expression between the pairwise comparisons. Genes with higher expression levels are located at the red edge while the genes with lower expression levels are located at the blue edge of the bar. Blue and red arrows indicated downregulation and upregulation, respectively, of the pathway in the pairwise comparisons. (**D**) Representative image and quantification of area of lipid droplets in brown adipose tissue. *Trf1^Δ/Δ^* had lower lipid droplets in brown adipose tissue. (**E**) Representative images and quantification of liver stained with F4/80 immunohistochemistry staining. Note that *Trf1^Δ/Δ^* presents higher macrophage infiltration. (**F**) Sirius red staining in the liver of 50-week- old females represented in images and quantification of the area of Sirius red staining. Note that 50 weeks of age *Trf1^Δ/Δ^* females have a higher area of Sirius red staining. (**G**) H2AX staining in the liver of RNAseq cohort of female mice. Note that *Trf ^Δ/Δ^* female mice have more DNA damage than wild-types. Error bars, s.e.m.; only significant values are shown; *P < 0.05; **P < 0.01; ***P < 0.001 determined by two-tailed Student’s *t*-test (**B**, **D**)

To gain a deeper understanding on the molecular mechanisms by which TRF1 can influence metabolism, we performed an RNA sequencing (RNA-seq) on the livers of fifteen weeks old *Trf1*^+/+^ and *Trf1^Δ/Δ^* female mice. This early time-point is previous to the differences in weight between genotypes (Figure 5A). Surprisingly, even at this early age, we observed increased liver weight, a phenomenon not previously noted in older mice, along with reduced mass of white adipose tissue in *Trf1^Δ/Δ^* mice compared to wild-type controls (Figure 5B). The higher liver weight in mice lacking Trf1 was unexpected, as all observed phenotypes thus far have been associated with improved metabolic aging and lower body weight. The decreased mass of white adipose tissue is consistent with the lower adiposity observed in the older cohort. Similarly, in line with these observations, fifteen weeks *Trf1*-deficient mice exhibited fewer fat droplets in their brown adipose tissue compared to wild-type controls. (Figure 5D).

Gene set enrichment analysis (GSEA) of *Trf1^Δ/Δ^* versus control *Trf1*^+/+^ RNA sequencing data showed that cholesterol biosynthesis and inflammatory pathways: inflammatory response, IL6-JAK-STAT3 signaling and TNFA signaling via NFKβ (Figure 5C) (Supplementary Figure 2A) were upregulated in *Trf1^Δ/Δ^* mice compared to wild-type mice. The upregulation of cholesterol biosynthesis was surprising; however, it explains the higher LDL serum plasma levels observed in *Trf1*-deficient mice. On the other hand, *Trf1* depletion has been described to cause inflammation [14, 25], in agreement with the upregulation of inflammatory pathways found here. In line with these findings, we observed increased macrophage infiltration as determined by F4/80 immunohistochemistry staining in liver *Trf1^Δ/Δ^* mice compared to the wild-type controls (Figure 5E). Furthermore, adiponectin, a hormone involved in glucose and fatty acid metabolism was found upregulated in the RNAseq data. On the other hand, we observed a significant downregulation of pathways related to myogenesis, the citric acid TCA cycle and respiratory electron transport, adipogenesis, oxidative phosphorylation and PPAR signaling in *Trf1^Δ/Δ^* mice compared to wild-type mice (Figure 5C). Hepatic stellate cells (HSCs) are quiescent liver-resident cells responsible for vitamin A storage and extracellular matrix (ECM) homeostasis. Upon liver injury or stress, HSCs become activated and transdifferentiated into a myofibroblast-like cells characterized by increased proliferation, contractility, and ECM production. The observed downregulation of myogenesis-related genes in the liver of mice lacking *Trf1*, suggests a shift from normal regenerative processes towards fibrotic remodeling, possibly driven by HSC activation in response to TRF1 abrogation. To test this hypothesis, we performed Sirius Red staining to assess liver fibrosis in the young female mice used for RNA-seq; no differences were observed at this early age. However, livers from fifty-week-old *Trf1^Δ/Δ^* female mice displayed a significantly increased Sirius Red-positive area compared to wild-type controls (Figure 5F).

To investigate the origin of the increased liver fibrosis, we assessed the burden of DNA damage in the livers of fifteen-weeks-old females mice using H2AX staining. We found that *Trf1* deficiency resulted in higher levels of DNA damage compared to wild-types controls (Figure 5G). A sustained DNA damage response following TRF1 depletion has been previously reported (Martínez et al., 2009; Bejarano et al., 2019).

Finally, other downregulated pathways were key metabolic pathways related with cellular energy production and metabolism regulation: oxidative phosphorylation, citric acid TCA cycle and respiratory electron transport, and PPAR pathway. These pathways provide insights into the observed phenotype of reduced weight and adiposity. Decrease expression of genes related to these pathways’ leads to a reduction in fat storage or deposition. Regarding energy storage, Periodic acid-Schiff (PAS) immunohistochemistry staining without and with diastase on the liver of fifteen-week-old females showed a tendency towards reduced glycogen content in *Trf1^Δ/Δ^* compared to wild-types (Supplementary Figure 2B).

In summary, young *Trf1*-deficient females displayed a higher liver weight, which was concomitant with an upregulation in pathways related to cholesterol biosynthesis and inflammation. We also found a downregulation on myogenesis pathways concomitant with increased DNA damage liver damage and fibrosis as determined by H2AX and Sirius red staining’s respectively. Major metabolic pathways related with energy production and regulation of metabolism homeostasis were also found downregulated in *Trf1*-deficient mice. In addition, white adipose tissue of *Trf1^Δ/Δ^* mice weight less compared to wild-types.

### Mice lacking TRF1 in the whole organism subjected to a high-fat diet show decreased body weight, lower adiposity and cholesterol levels

**Figure 6:**
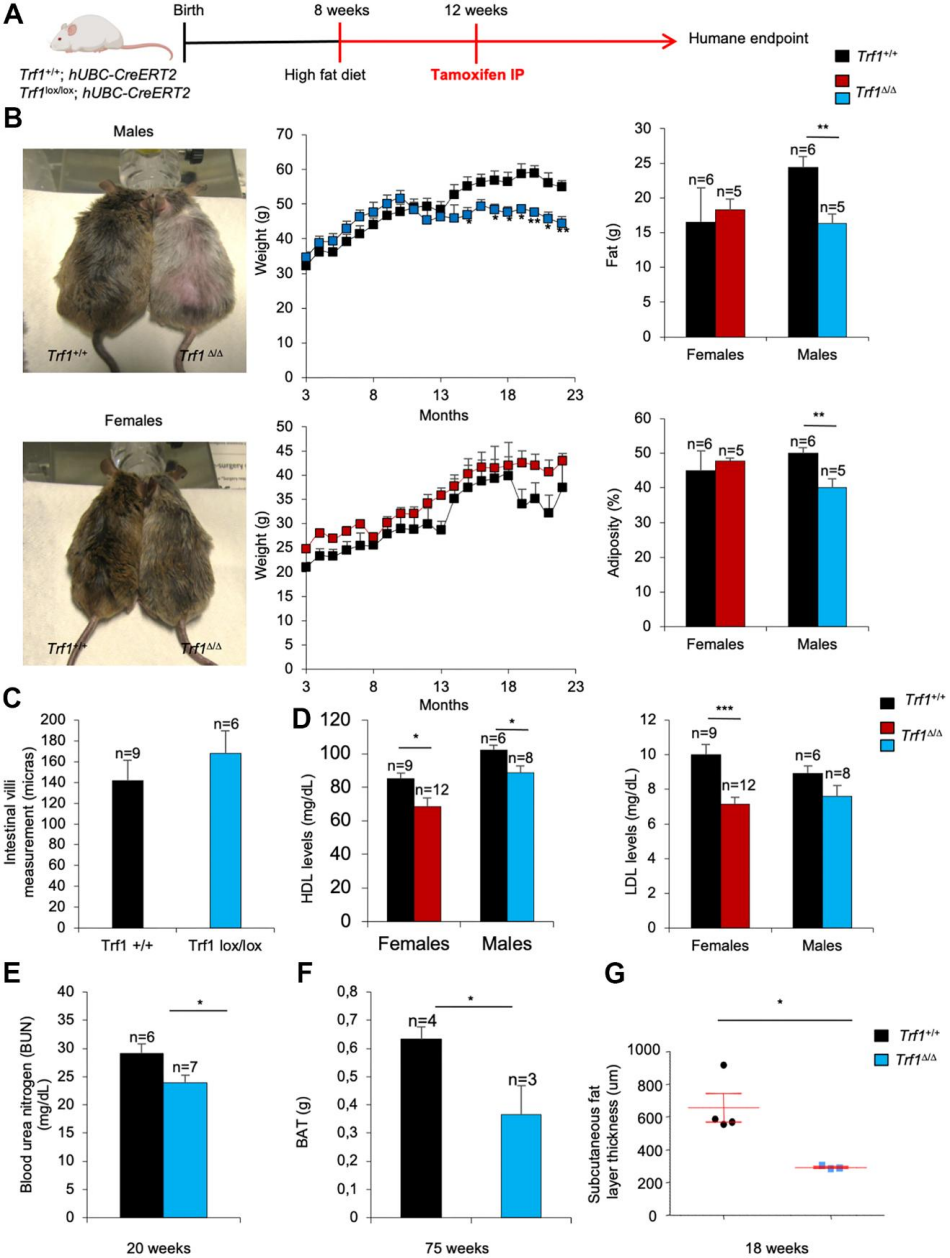
**TRF1 ablation leads to weight loss and less adiposity in high-fat diet males but not in females.** (**A**) Experimental plan: *Trf1*^+/+^ and *Trf1*^lox/lox^; *hUBC-CreERT2* subjected to a high-fat diet at 8 weeks and treated intraperitoneally with tamoxifen at 12 weeks until humane endpoint. (**B**) Representative images of males and females at 17 months (left), weight (center), fat (g) and percentage of adiposity measured by DEXA (right). Note that *Trf1^Δ/Δ^* males weigh less and have lower fat content and adiposity. (**C**) Length measurement of intestinal villi (microns). (**D**) HDL and LDL levels (mg/dL) in serum 18-week-old mice of *Trf1*^+/+^ and *Trf1^Δ/Δ^* in both sexes. (**E**) Measurement of blood urea nitrogen (BUN) in plasma of male mice. (**F**) Weight of brown adipose tissue (BAT) of males. (**G**) Subcutaneous fat layer thickness (um) of males. Error bars, s.e.m.; only significant values are shown; *P < 0.05; **P < 0.01; ***P < 0.001 determined by two-tailed Student’s *t*-test (**B**–**G**)

Next, we addressed the impact of a high-fat diet in the metabolic phenotypes found in TRF1-abrogated mice. Under a high-fat diet, *Trf1^Δ/Δ^* males showed a significant lower body weight, lower total fat content, and lower adiposity compared to wild-type male controls, while this was not observed in female counterparts (Figure 6A). Again, the decreased body weight in males subjected to a high fat diet was not associated to intestinal atrophy as the consequence of TRF1 abrogation as indicated by similar length of the intestinal villi in *Trf1^Δ/Δ^* and wild-type males (Figure 6B).

At 18 weeks of age both *Trf1^Δ/Δ^* females and males subjected to a HFD showed significantly lower levels of HDL compared to wild-type controls (Figure 6C). Similarly, LDL cholesterol levels were lower in *Trf1^Δ/Δ^* mice of both genders, but this difference only reached statistical significance in *Trf1^Δ/Δ^* females (Figure 6C).

Furthermore, *Trf1^Δ/Δ^* males showed statistically significant lower levels of blood urea nitrogen (BUN) in plasma compared to wild-type controls (Figure 6D), indicating lower kidney damage.

The weight of the brown adipose tissue was also significantly decreased in *Trf1^Δ/Δ^* males compared to wild-type controls (Figure 6E). In addition, the subcutaneous fat layer thickness was strikingly thinner in *Trf1* deleted males compared to wild-type counterparts (Figure 6F). Together, these findings suggest that *Trf1* plays a role in modulating body weight by reducing fat content.

### Mice lacking TRF1 in the whole organism subjected to a high-fat diet show improved glucose metabolism

**Figure 7:**
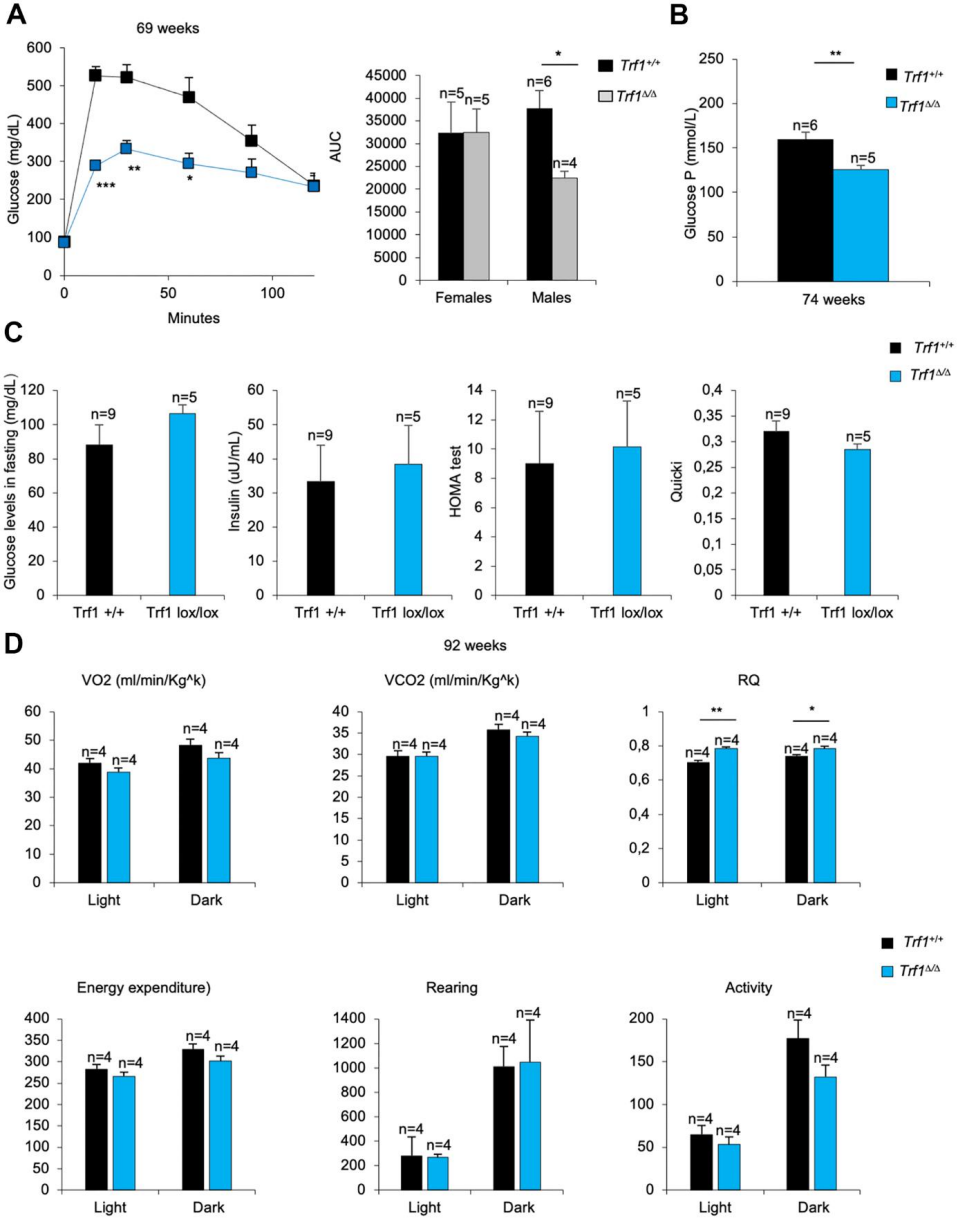
**TRF1 deleted males’ better tolerance to glucose.** (**A**) Glucose tolerance test: glucose levels (mg/dl) in males and quantification of the area under the curve (AUC) in females and males. Note that males without *Trf1* fed with high-fat diet have better tolerance to glucose. (**B**) Plasma glucose P levels in males. (**C**) Males fed in high-fat diet glucose (mg/dL) and insulin levels (uu/mL) in fasting. Derived HOMA-IR insulin resistance quantification, and QUICKI insulin sensitivity quantification in males. (**D**) Volume of oxygen consumed (VO2), volume of carbon dioxide produced (VCO2) (ml/min/Kg^k^), respiratory quotient (RQ), energy expenditure rate (EE) (Kcal/day/Kg^k^), activity and rearing monitorization in 92 weeks males’ mice. Error bars, s.e.m.; only significant values are shown; *P < 0.05; **P < 0.01; ***P < 0.001 determined by two-tailed Student’s *t*-test (**A**–**D**)

To explore whether glucose metabolism was also improved in *Trf1^Δ/Δ^* mice subjected to a high-fat diet, we performed a glucose tolerance test. Males without *Trf1* showed a better tolerance to glucose compared to wild-type controls (Figure 7A), however this difference was not observed in similarly treated females (data not shown).

To further study the male phenotype, we studied glucose and insulin metabolism. Plasma glucose P levels were strikingly lower in *Trf1* deleted mice compared to wild-type males (Figure 7B). When mice were subjected to fasting, glucose and insulin levels were similar between genotypes. In accordance with mice fed with high-fat diet, both genotypes showed resistance to insulin (Figure 7C). Next, we measured the Respiratory Quotient (RQ) in both genotypes by using metabolic cages. An RQ between 0.7 and 1 indicates that the sources of energy are protein and fat. In high-fat fed mice, the expect RQ should be under 0.7, as fat is the main source of energy. As expected, wild-type males showed an RQ below 0.7 (Figure 7D). In contrast, the RQ in *Trf1*-deficient mice was close to 0.7 (Figure 7D), suggesting that they use protein and fat as energy sources. No differences were observed in VO2, VCO2, energy expenditure, rearing and activity (Figure 7D). Food intake could not be determined as the mice were gnawing the food.

Next, we performed full histological analysis of both male and female of both genotypes mice subjected to high-fat diet mice. Fewer and smaller lipid droplets were observed in liver, white adipose tissue and brown adipose (Supplementary Figure 2C). Again, the *Trf1^Δ/Δ^* mice exhibited a significantly larger H2AX-positive area relative to the total area compared to the wild-type controls. (Supplementary Figure 2D).

Finally, we observed that both *Trf1* deficient male and female mice subjected to both normal and high-fat diets had lower fat accumulation in visceral and gonadal areas compared to wild-type controls at time of death (Supplementary Figure 2E).

These results suggest that *Trf1* abrogation may impede the utilization of fat as main energy source, probably as the consequence to the reduced content of white adipose tissue.

## DISCUSSION

Previous research has suggested a protective role for telomeres in preventing obesity [18, 20]. In particular *Tert-*deficient mice in adipocyte progenitors predisposes to metabolic disease [18] while mice with hyper-long telomeres are significantly leaner and show improved glucose and insulin tolerance [20]. Similarly, our group and others have previously shown a role of the RAP1 shelterin in protecting from obesity and metabolic syndrome [21, 22]. However, a deeper understanding of the role of the shelterin complex in metabolism is largely unknown.

In this regard, a human study previously identified of the TRF1 shelterin protein as a regulator of telomere attrition in obese patients [23]. In line with a potential connection between TRF1 and obesity, we found that mice lacking the TRF1 telomere binding protein showed persistent weight reduction, loss of fat content, and improved glucose and insulin tolerance independently of telomere length. The lower fat content in *Trf1^Δ/Δ^* mice was confirmed by DEXA, including reduced weight of both white and brown adipose tissues, decreased lipid droplets in the brown adipose tissue, a thinner subcutaneous fat layer, as well as reduced presence of triglycerides in blood with age. Of interest, the decreased body weight phenotype was observed in male but no female mice when subjected to a high-fat diet, suggesting a potential hormonal effect. Female mice are often not included in high-fat diet studies because they tend to have greater protection against diet-induced obesity compared to male mice [26, 27].

Given the previously known roles of TRF1 in telomere protection and prevention of a persistent DNA damage response at telomeres [28, 29], as well as in the maintenance of adult stem compartments and tissue homeostasis [14], we first discarded intestinal atrophy as the potential cause of leanness in *Trf1^Δ/Δ^* mice. We also excluded higher activity or lower food intake as the cause of the phenotypes in *Trf1*-deleted mice. Of interest, fifty-week-old females had higher metabolic activity as evidenced by higher VCO2, energy expenditure, and food intake. Moreover, *Trf1*-deficient males had a respiratory quotient (RQ) of approximately 0.8 on both normal and high-fat diets, suggesting they used both fat and protein as energy sources. This is interesting because mice fed with a high-fat diet, tend to have a RQ around 0.7, corresponding to fat being the main energy source. The finding that *Trf1* mice need the use of another energy source together with fat, could be attributed to lower fat content or a deficient capacity of lipid absorbance or storage. This hypothesis agrees with their reduced adiposity and fat content. We also found an improved glucose metabolism in *Trf1^Δ/Δ^* mice in both the normal and the high fat diet. Finally, glycogen storage in the liver was lower in *Trf1* deficient mice compared to wild-type controls.

As mentioned above, a previous clinical study described upregulation of TRF1 in obese patients as a potential major contributor to short telomeres in these patients [23]. However, we did not observe any differences in telomere length between the genotypes, suggesting that the lean phenotype observed here is independent of telomere length changes induced by TRF1 protein depletion.

RNA sequencing data revealed that *Trf1* depletion triggers a cascade of inflammatory responses and upregulates cholesterol biosynthesis pathways in the liver, which is consistent with the observed increase in macrophage infiltration in liver of *Trf1^Δ/Δ^* mice. While chronic inflammation is typically associated with metabolic dysfunction and obesity, the distinct phenotype observed in Trf1-deficient mice, which is characterized by increased inflammation and reduced adiposity, suggests a potential novel role for TRF1 in modulating the interplay between inflammatory and metabolic pathways. Specifically, elevated NF-κB activity has been shown to increase energy expenditure and prevent adiposity, possibly through upregulation of pro-inflammatory cytokines like TNF-α and IL-6, which are known to promote energy expenditure and protect against obesity and insulin resistance [30, 31]. These findings raise the possibility that TRF1 may act as a regulatory node that balances inflammatory signaling in a manner that favors energy expenditure over metabolic dysfunction. In this context, TRF1 loss may promote a catabolic state, contributing to lower fat accumulation and reduced body weight. Further studies are needed to elucidate the mechanistic underpinnings of TRF1’s protective role in regulating the balance between inflammation and energy metabolism. We also observed downregulation of genes related to important metabolic pathways such as oxidative phosphorylation, electron transport chain, myogenesis, the citric acid TCA cycle, respiratory electron transport, adipogenesis, and PPAR signaling pathways in *Trf1^Δ/Δ^* mice compared to wild-type animals. Specifically, the downregulation of the citric acid (TCA) cycle and respiratory electron transport pathways could indicate impaired mitochondrial function and reduced energy production, leading to decreased energy storage and fat deposition. Additionally, the downregulation of adipogenesis is directly correlated with reduced fat formation, explaining the lower fat content observed in these mice. In agreement with this notion, adiponectin, a hormone synthetized in adipose tissue and involved in the metabolism of fatty acids and glucose, was found to be upregulated in the liver of *Trf1^Δ/Δ^* mice. A previous study also described an inverse correlation between adiponectin levels and fat mass [32], in agreement with our findings. On the other hand, the observed decrease in oxidative phosphorylation may result in less effective ATP production, which in turn may lead to a higher catabolic rate to generate energy to meet demands and reduce the fat storage. Finally, we found lower expression of PPAR signaling, known to regulates lipid and energy balance, which may result in reduced lipid uptake and storage in adipose tissue leading to diminished lipid storage. Together, these findings are in agreement with the reduced weight and lower fat phenotypes in mice lacking *Trf1*.

Deficient lipid storage and reduced fat content may also be the consequence of TRF1’s role in stemness [14, 33–35]. TRF1, known for its role in telomere maintenance and stem cell protection, could be essential for the proper functioning of the adipose tissue progenitor pool. Its depletion may compromise adipose stem cell proliferation and differentiation, thereby reducing the generation of new adipocytes and the fat storage capacity. Specifically, fat is produced through the induction of hypertrophy and hyperplasia, and TRF1 depletion may impair fat stem cells, leading to decreased fat storage. Supporting this hypothesis, RNA sequencing data, as previously mentioned, revealed a downregulation of key adipogenesis-related pathways, including PPAR signaling, as well as genes associated with mitochondrial function, oxidative phosphorylation, and the electron transport chain, indicating impaired energy production and adipose progenitor functionality.

Finally, we found that the liver of the *Trf1^Δ/Δ^* female mice weighted more than normal. It has been previously reported that TRF1 depletion causes DNA damage and inflammation [14, 25]. Similarly, young female *Trf1^Δ/Δ^* livers showed increase of inflammation, increased fibrosis and increased DNA damage. These findings maybe the consequence of decreased liver regeneration as the consequence of TRF1 deficiency in agreement with its known role in stemness. Nevertheless, these liver phenotypes were very mild and not accompanied by abnormal liver histology, concomitant with normal levels of ALT, ALP and albumin, which are markers of liver damage.

In summary, we describe here that decreased fat content is the main cause of weight reduction associated to TRF1 abrogation together with a better glucose metabolism. These findings reveal a previously unknown role of TRF1 in the regulation of metabolism.

## MATERIALS AND METHODS

### Mouse models

*TRF1^lox/lox^* mice [29] were crossed with the mouse strain carrying ubiquitously expressed, tamoxifen activated recombinase, *hUBC-CreERT2* [36] to generate TRF1*^+/+^* or TRF1*^lox/lox^*; hUBC-CreERT2 mice. All mice have been maintained at the Spanish National Cancer Centre under specific pathogen-free conditions, with a 12h light-dark cycle in a temperature-controlled room following the recommendations of the Federation of European Laboratory Animal Science Associations (FELASA). All the experiments were approved by the CNIO-ISCIII Ethics Committee and, by Consejería de Medio Ambiente, Administración Local y Ordenación del Territorio (Comunidad de Madrid). All experiments have been performed in accordance with the guidelines for ethical conduct in the care and use of animals as stated in the international guiding principles for biomedical research involving animals, developed by the Council for International Organizations of Medical Sciences (CIOMS). Littermates of the same sex were randomly assigned to either experimental or control groups. Developmental stage of mice is included appropriately in the text and figure legends. Food (Harlan Laboratories and Research Diets) and water were provided ad libitum, unless otherwise noted. The timepoints were selected according to the health status of the mice and the required recovery periods between experiments. This approach helped minimize stress on the animals and ensured stable conditions for the tests.

### Mouse diets

Mice were randomized into test groups and given ad libitum access to either a chow (18% fat, 58% carbohydrates and 24% proteins) (Harlan Laboratories, 2018S) or high-fat diet (45% fat, 35% carbohydrates and 20% proteins) (Research Diets, D12451) at the age of 10 weeks and, 8 weeks in the case of high-fat diet fed mice. Cre-mediated recombination was activated by tamoxifen intraperitoneal injection at 10 weeks and, 12 weeks for high-fat diet mice.

Intraperitoneal injection of tamoxifen was administrated (1mg/injection) 3 times per week for 1-1,5 month and then 1 boost every 15 days. Body weight were measured every week.

### Blood parameters

Blood was collected from the facial vein to an EDTA tube for hemogram analysis using Laser Cell. Metabolic parameters such as ALT, ALP, total bilirubin, creatinine, total cholesterol, albumin and blood urea nitrogen were quantified by Vetscan mammalian liver profile. Plasma was obtained after 4000g, 20 minutes centrifugation. TGC (triglycerides), glucose P, HDL and LDL levels were determined by ABX Pentra (Horiba Medical). Insulin was measured using ultrasensitive mouse insulin ELISA kit (Christal chem ELISA). HOMA-IR was calculated following the formula: fasting insulin [F06DU/mL] x fasting glucose [mg/dL]/22.5. Quicki formula followed was: 1/[log (fasting glucose [mg/dl]) + log (fasting insulin [μU/mL]). The conversion factor used for calculate HOMA and Quicki given by Christal chem ELISA was: 1ng/mL = 24 μIU/mL.

### Body composition analysis

Animals were anaesthetised with 2% isoflurane (Isovet, Braun Vetcare) and total fat, lean and fat masses were measured by Dual energy X-ray Absorptiometry (DEXA) (PIXImus, Lunar Corporation). The analysis was performed using a region of interest (ROI) in the whole body using Lunar PIXImus 2.10 software.

### Gene expression analysis

Total RNA from tissue was extracted with the RNeasy kit (QIAGEN). For RNA-seq experiments, total RNA samples were used. RNA quality scores were 7,51 on average assayed by a PerkinElmer LabChip analyzer. For library generation, reverse transcription using oligo-dT priming was performed and subsequently, a second-strand synthesis was performed using random primers and a DNA polymerase. Both sets of primers included Illumina-compatible sequences. The resulting cDNA libraries were purified and applied to an Illumina flow cell to generate clusters. Sequencing was carried out on an Illumina NextSeq 550 using v2.5 reagent kits, following the manufacturer’s protocols. The reads underwent analysis using a Snakemake-based pipeline developed at the CNIO Bioinformatics Unit (https://github.com/cnio-bu/cluster_rnaseq). The quality of sequencing was assessed using FastQC v0.11.9, and absence of sample contamination from other species was verified with FastQ Screen v0.15.1. To remove sequencing adaptors and poly(A) tails, the ‘bbduk.sh’ command was applied as per Lexogen recommendations. Alignment of the processed reads to the mouse reference genome (GRCm39) was performed using STAR v2.7.10a, and read counts were obtained using featureCounts [37]. For gene annotation, GENCODE GRCm39 vM27 was used.

Differential expression was done with DESeq2 [38], and those genes with an FDR < 0.05 were considered as significant.

Functional analysis was performed with Gene Set Enrichment Analysis (GSEA) employing 1,000 gene-set permutations only gene sets exhibiting significant enrichment levels (FDR q-value < 0.25) were considered.

### Glucose tolerance test and insulin tolerance test

For GTT, mice were fast for 12 hours and injected intraperitoneally 2g of glucose/kg of body weight in PBS. For ITT, 0,75U insulin /kg of body weight in PBS (Humalog Insulin) was intraperitoneally administered. Glucose levels were measured from the tail vein before, and at 15, 30, 60, 90 and 120 minutes after the injection using a glucometer.

### Immunohistochemistry analyses in tissue sections

Tissues were fixed overnight in 10% formalin solution (sigma) and embedded in paraffin. For immunohistochemistry staining, tissue sections were deparaffinized with 10mM sodium citrate for 2 minutes. Slides were washed in water, then in buffer TBS Tween20 0,5%, blocked with peroxidase, washed again with TBS Tween 20 0,5% and blocked with fetal bovine serum followed by another wash.

### Primary antibodies

Hematoxylin and eosin (H&E) staining was performed for the quantification of intestinal villi and subcutaneous skin fat layer. H&E staining was also employed to evaluate tissue morphology and integrity of liver, white and brown adipose tissue, pancreas, spleen, intestine, colon, kidney, heart, lung, brain and muscle. Sirius red staining was used for determining liver fibrosis. H2AX staining was utilized for the evaluation of DNA damage in liver. Periodic acid-Schiff staining without and with diastase was performed to detect the presence of glycogen storage. Followed by incubation with secondary antibodies conjugated with peroxidase from DAKO.

Olympus AX70 microscope was used for taking pictures of immunohistochemistry tissue samples. The percentage of positive cells was identified using ZEISS ZEN 3.7 software.

### Metabolic activity

Metabolic activity was determined using Oxylet system metabolic chambers (Panlab Harvard Apparatus). Mice were individually caged for 1 week for acclimation. During the 72-hour measurement period, the mice were provided unrestricted access to food and water. Throughout the entire study duration, mouse activity, food intake, and drink intake were observed and recorded at 2-minute intervals. The respiratory exchange ratio (RQ) was calculated by dividing the volume of consumed O2 (VO2) by the volume of eliminated CO2 (VCO2), which were measured every 24 minutes. Energy expenditure (EE) was determined using the formula EE = (3.815 + (1.232 × RQ)) × VO2 × 1.44. The data analysis was conducted using Metabolism 3.0 software.

### Telomere length analysis in tissue sections

To analyze telomere length, a quantitative telomere fluorescence *in situ* hybridization (qFISH) was performed. Tissues were embedded in paraffin and were deparaffinized. Followed first by a digestion with pre-warmed pepsin/HCl and then 4% formaldehyde for fixation. Slides were dehydrated by increasing concentration of ethanol (70%, 90%, and 100%). Next, the samples were incubated with the telomeric probe for 3.5 minutes at 85°C followed by another incubation for two hours at room temperature in a wet chamber. The slides were washed extensively with 10mM Tris in 70% formamide and with 0,08% TBS-Tween. DAPI was added and the slides were mounted with Vectashield. Immunofluorescence images were obtained using a CS SP8 STED 3X (Leica-Microsystems) under the Navigator software integrated in the LAS X v3.5.7 (Leica-Microsystems).

A robust methodology was developed to identify and characterize telomere signals using Cellpose [39] algorithm, which leverages deep learning techniques. Our approach consisted of three key components: telomere/nucleus identification, telomere-nucleus pairing, feature extraction and characterization to assess telomere-nucleus interactions. The model for telomere segmentation was fine-tuned on our specific dataset to enhance performance, then telomeres were accurately delineated, providing precise localization and segmentation results. In parallel, nuclei segmentation was crucial for associating telomeres with their respective cellular context. In this regard, a custom pipeline in Groovy, utilizing ImageJ libraries, linked each telomere to its corresponding nucleus, establishing telomere-nucleus pairs. This pairing process was meticulously validated for accuracy and consistency. Subsequently, for each telomere-nucleus pair, telomere feature extraction and characterization were computed to quantify telomere fluorescence signal strength, size and spatial distribution with their corresponding descriptive statistics to assess telomere quantification. To determine telomere localization, fluorescence intensity distribution was analyzed in telomeres across all cells in our dataset. Then, positive thresholds were computed based on mean intensity and deviation from the average. Telomeres with fluorescence marker intensities significantly deviated from the norm were classified as positive.

### Statistical analysis

Survival data was analyzed by Kaplan Meier survival curves, and comparisons were conducted via the Log Rank test. The statistical analysis was assessed through the use of GraphPad Prism 10.1.1 software. Quantification of immunohistochemistry was carried out using ZEISS ZEN 3.7 software, while telomere length was determined through the application of the Cellpose algorithm as previously detailed in earlier sections. Unpaired Student’s t-test was used to determine statistical significance, with P-values below 0.05 considered as significant. The statistical analysis was executed using Microsoft Excel 16.66.1.

### Data availability

The RNA-seq data has been archived in the Gene Expression Omnibus of the National Center for Biotechnology Information under the accession number GSE273596.

## Supplementary Material

Supplementary Figures
