# Supplementary material for: Depletion of the TRF1 telomere-binding protein leads to leaner mice with altered metabolic profiles
Source: Aging (Albany NY). 2025 Sep 17;17(9):2410–29. doi: 10.18632/aging.206320 (PMC12517213; doi:10.18632/aging.206320)
Supplement: Supplementary Figures [file aging-17-9-206320-s001.pdf]

SUPPLEMENTARY FIGURES

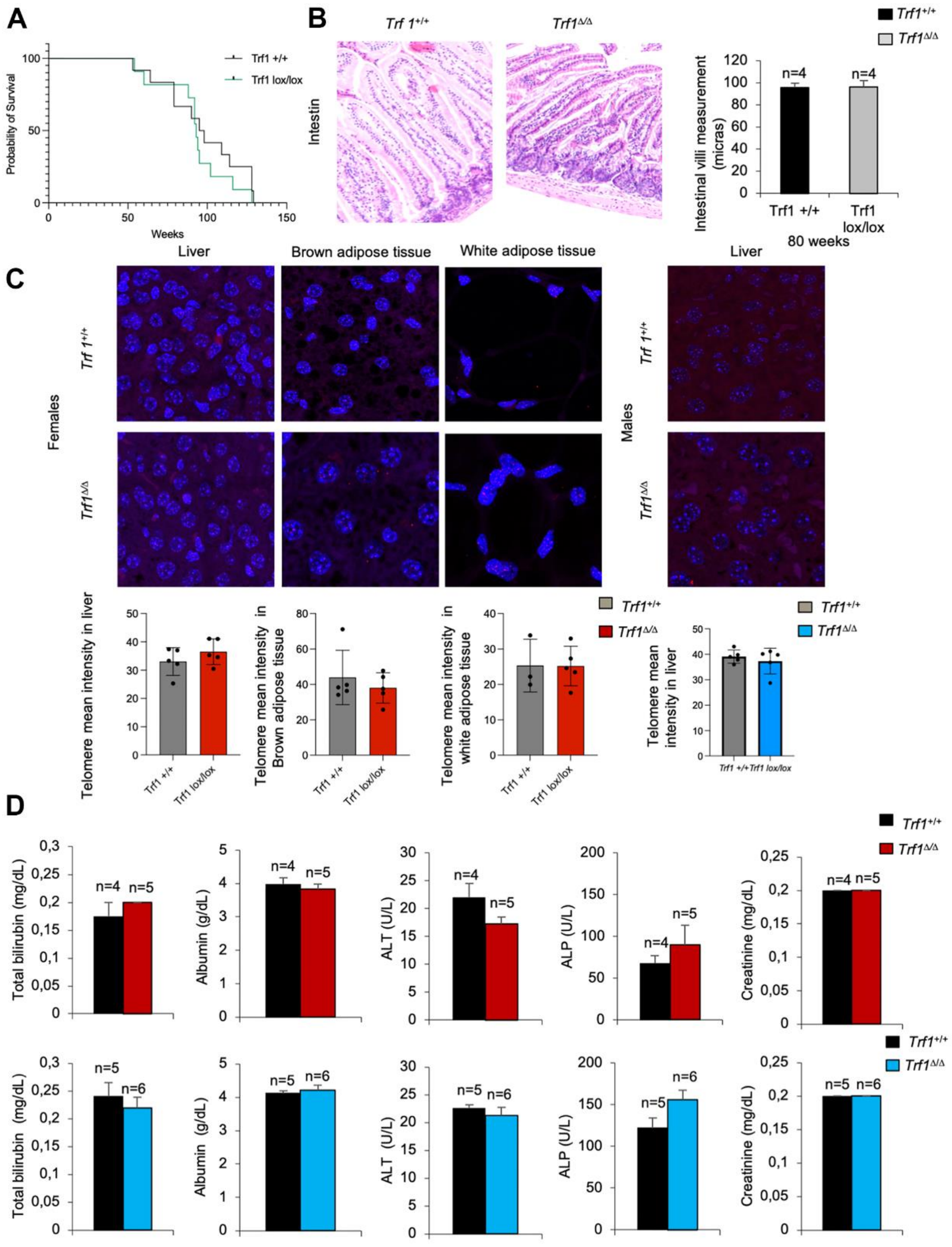

Supplementary Figure 1. (A) Survival curve analysis of *Trf1*$^{+/+}$ and *Trf* $^{\Delta/\Delta}$ mice. Note that survival is similar between genotypes. (**B**) Intestinal villi measurement. Left: representative images of intestine villi stained with hematoxylin and eosin. Right: quantification of intestinal villi measurement (microns). Note that no differences are observable. (**C**) Telomere length measurement in the liver, brown adipose tissue, and white adipose tissue. Females on the left and males on the right. Telomere length in adipose tissue could not be measured in males. Note similar telomere length between genotypes. (**D**) Blood chemistry measurement of *Trf1*$^{+/+}$ and *Trf1*$^{\Delta/\Delta}$ mice. Total bilirubin, albumin, alanine aminotransferase, phosphatase alkaline, and creatinine. Note that no differences are observable. Error bars, s.e.m.; only significant values are shown; *$P < 0.05$; **$P < 0.01$; ***$P < 0.001$ determined by Kaplan Meier and two-tailed Student's *t*-test (**A**–**C**)

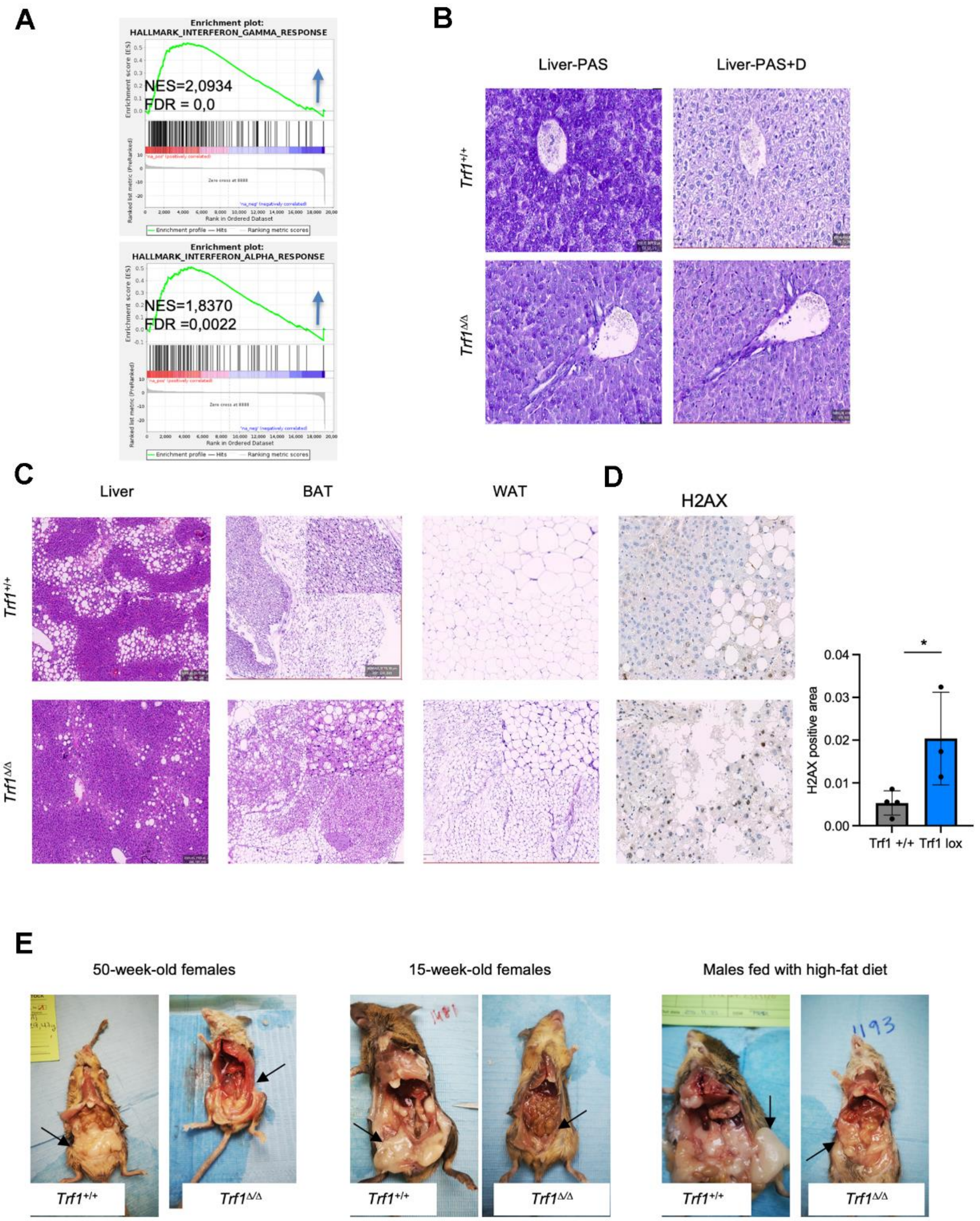

Supplementary Figure 2. (A) Gene expression data obtained by RNA-seq of liver samples of 15 weeks *Trf1*$^{+/+}$ and *Trf1*$^{\Delta/\Delta}$ was analyzed by GSEA to determine significantly enriched gene sets. GSEA plots for the inflammatory pathways in liver samples for *Trf1*$^{\Delta/\Delta}$ versus *Trf1*$^{+/+}$ mice. The red to blue horizontal bar represents the ranked list. Genes located at the central area of the bar show small differences in gene expression between the pairwise comparisons. Genes with higher expression levels are located at the red edge while the genes with lower expression levels are located at the blue edge of the bar. Blue and red arrows indicated downregulation and upregulation, respectively, of the pathway in the pairwise comparisons. (**B**) Periodic acid-Schiff staining without and with diastase in the liver of 15 weeks *Trf1*$^{+/+}$ and *Trf1*$^{\Delta/\Delta}$ female's mice. Note that in *Trf1*$^{+/+}$mice there is a change when diastase is present, from purple without diastase to light violet when the staining is with diastase. However, this is not the case for mice without *Trf1.* (**C**) Hematoxylin and eosin staining of liver, white and brown adipose tissue of males fed with high-fat diet. Note that fewer and smaller lipid droplets are observed in liver, brown adipose tissue and white adipose tissue in *Trf1*$^{\Delta/\Delta}$ compared to wild-types. (**D**) H2AX staining for measuring DNA damage in the liver of male's mice fed with a high-fat diet. Note that there is more DNA damage in mice without *Trf1* than in the wild-types. Error bars, s.e.m.; only significant values are shown; *$P < 0.05$; **$P < 0.01$; ***$P < 0.001$ determined by two-tailed Student's *t*-test (**C**). (**E**) Representative images of necropsies of mice. On the left are 50-week-old females, at the center are males fed with a high-fat diet, and on the right are 15-week-old female mice. Note that, *Trf1*$^{\Delta/\Delta}$ mice contain less fat content compared to wild-types (black arrows).
